# RAM is upregulated during T cell activation and is required for RNA cap formation and gene expression

**DOI:** 10.1093/discim/kyad021

**Published:** 2023-11-17

**Authors:** Katarzyna Knop, Carolina Gomez-Moreira, Alison Galloway, Dimitrinka Ditsova, Victoria H Cowling

**Affiliations:** Cancer Research UK Scotland Institute, Glasgow, G61 1BD, UK; School of Life Sciences, University of Dundee, DD1 5EH, Dundee, UK; School of Life Sciences, University of Dundee, DD1 5EH, Dundee, UK; Cancer Research UK Scotland Institute, Glasgow, G61 1BD, UK; School of Life Sciences, University of Dundee, DD1 5EH, Dundee, UK; Cancer Research UK Scotland Institute, Glasgow, G61 1BD, UK; School of Life Sciences, University of Dundee, DD1 5EH, Dundee, UK; Cancer Research UK Scotland Institute, Glasgow, G61 1BD, UK; School of Life Sciences, University of Dundee, DD1 5EH, Dundee, UK; School of Cancer Sciences, University of Glasgow, G61 1QH, Glasgow, UK

**Keywords:** T cells, transcriptomics, activation, RNA cap, transcription, translation

## Abstract

On T cell activation, upregulation of gene expression produces the protein required for the differentiation and proliferation of effector cell populations. RAM (RNMT-Activating Mini protein/RAMAC/Fam103a1), the cofactor of the RNA cap methyltransferase RNMT (RNA guanosine N-7 cap methyltransferase), is upregulated following activation. Formation of the RNA cap protects RNA during synthesis and guides RNA processing and translation. Using conditional gene deletion, we found that *Ram* expression stabilizes RNMT protein in T cells and is required for its upregulation on activation. When the *Ram* gene is deleted in naïve T cells, there are major impacts on activation-induced RNA cap formation and gene expression. Activated T cell proliferation is dependent on increased ribosome production; in *Ram* knockout T cells, activation-induced expression of ribosomal protein genes and snoRNAs is most severely reduced. Consistent with these changes, *Ram* deletion resulted in reduced protein synthesis, and reduced growth and proliferation of CD4 T cells. Deletion of *Ram* results in a similar but milder phenotype to *Rnmt* deletion, supporting the role of RAM as a RNMT cofactor.

## Graphical Abstract

**Graphical Abstract:**
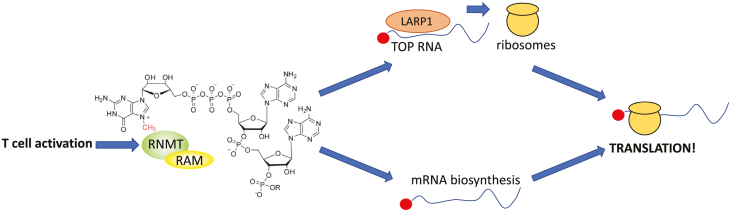

## Introduction

When T cells are activated in response to antigen recognition, elevated transcription, RNA processing and translation accelerate the protein production required for rapid proliferation and differentiation into effector lineages [1–4]. During this phase of rapid gene expression, increased RNA cap formation on RNA pol II transcripts is critical [5]. The RNA cap protects RNA during transcription and recruits factors involved in RNA processing, export, and translation [6, 7]. Upregulation of the major RNA cap methyltransferase, RNMT (RNA guanosine N-7 cap methyltransferase), is required for increasing gene expression in activated T cells [5].

RNMT increases mRNA production via co- and post-transcriptional stabilization of RNA and via non-catalytic impacts on mRNA production [5, 7–9]. A key function of the RNA cap is to mediate the recruitment of mRNA to the ribosome. In T cells, RNMT additionally increases the ribosome content. RNMT has specific impacts on ribosome production by selectively upregulating RNAs encoding ribosomal proteins, ribosomal RNA synthesis and processing factors, and ribosome biogenesis factors [5]. Many RNAs governing ribosome production or translation are members of the TOP RNA family, which have a 5ʹ polypyrimidine tract that binds to the RNA cap-binding protein LARP1 (La Ribonucleoprotein 1, Translational Regulator), which protects RNA from degradation and impacts on translation [10, 11]. *In vitro*, RNMT-catalysed N-7 cap guanosine methylation is required for LARP1 to interact with capped RNA and in T cells LARP1-interacting RNAs are repressed following *Rnmt* gene deletion [5, 12–14]. Thus, the LARP1–^m7^G interaction selectively enhances the stability of TOP-RNAs during T cell activation, providing the increased ribosome content required for RNA translation and cell proliferation.

The expression and activity of RNMT are dependent on its cofactor RAM (RNA-activating mini protein/RAMAC) [15]. RAM has several positive impacts on RNMT function; RAM binding to RNMT alters active site dynamics resulting in increased RNA cap methylation, it has a RNA binding domain which recruits RNA to the RNMT complex, and it increases the stability of RNMT [8, 15–18]. During embryonic stem cell differentiation, regulation of RAM has a critical role [19, 20]. RAM is repressed during embryonic stem cell differentiation resulting in reduced RNMT activity; this mechanism is necessary for the repression of pluripotency genes. Conversely, RAM is upregulated during T cell activation, leading to questions about the role of RAM in gene regulation during this process [5].

Here we report that during T cell activation, RAM is required for the upregulation of RNMT and the gene regulation associated with ribosome production. Deletion of the *Ram* gene results in reduced protein synthesis and reduced proliferation in activated T cells.

## Materials and methods

### Mice

Animal experiments were performed in accordance with UK Home Office and ARRIVE guidelines. Reviewed by the University of Dundee Welfare and Ethical Use of Animals Committee and the University of Glasgow Animal Welfare and Ethical Review Board. *Ram (Ramac/Fam103a1)*^fl/fl^ mice (loxP sites flanking exon 2 of *Ramac)* were sourced from Taconic Artemis Gmbh. *Rnmt*^fl/fl^ mice with loxP sites flanking exon 3 of *Rnmt* were published previously [5]. CD4-Cre (Tg(Cd4-cre)1Cwi) mice were published previously [21]. Animals used in this study were on a C57BL/6J background. Mice were housed in a pathogen-free environment and kept under standard conditions with a 12-h day/night cycle with access to food and water *ad libitum*. Environmental enrichments were added to all cages. Genotyping of ear notches taken at weaning was performed by either the MRC unit genotyping team or Transnetyx. Mice were bred in compliance with EU and Health Products Regulatory Authority standards and used between 8 and 14 weeks old. For all experiments, one mouse represents one biological replicate.

### T cell extraction

Lymph nodes (inguinal, brachial, axillary, superficial cervical, mesenteric, lumbar, and caudal), spleens, and thymi were dissected from mice and passed through a 70 µm cell strainer (Falcon) to prepare cell suspensions in T cell culture medium: RPMI 1640 medium, Thermo Fisher Scientific) supplemented with 10% heat-inactivated Fetal Bovine Serum (FBS) (Thermo Fisher Scientific), 50 000U of Penicillin–Streptomycin (Thermo Fisher Scientific) and 50 µM B-mercaptoethanol (Merck). Cells were counted either using the BD FACSVerse (BD Biosciences), Novocyte (Acea Biosciences) or Attune NxT Flow Cytometer (Thermo Fisher Scientific). For this purpose, cells were incubated with labelled antibodies and FC block (anti-CD16/32, Biolegend) in Phosphate-buffered Saline (PBS) + 2% FBS. Cells were labelled with 0.1 μg/ml DAPI. Details of all antibodies used in this study are in Supplementary Table S6.

### *Ex vivo* activation of CD4 and CD8 T cells

For proliferation analysis, cells isolated from spleens were cultured in T cell culture medium with 0.5 μg/ml Ultra-LEAF purified anti-mouse CD3ε antibody (clone 145-2C11, Biolegend), and 0.5 μg/ml Ultra-LEAF purified anti-mouse CD28 antibody (clone 37.51, Biolegend). 20 ng/ml interleukin 2 (IL-2, Proleukin, Norvatis) was added on days 2 and 3. CD4 or CD8 T cell numbers and forward scatter (FSC-A) were assessed using a Novocyte Flow Cytometer (Aceabio) or Attune NxT Flow Cytometer (Thermo Fisher Scientific). CD4 T cells were MACS magnet sorted using a mouse CD4 + T cell Kit (130-104-454; Miltenyi Biotec). Live and T cell numbers and purity (resulting in a minimum of 90%) were assessed using a Novocyte flow cytometer (Aceabio). Gating strategies for FACS are presented in Supplemental Figs. 1–4.

### RNA preparation for mass spectrometry and RNA sequencing

RNA was extracted using Tri-reagent (Sigma/Merck) or Trizol (Thermo Fisher Scientific) and quantified by Nanodrop (Thermo Fisher Scientific) or Qubit™ RNA BR Assay Kit (Thermo Fisher).

### CAP–MAP

Cap analysis protocol with minimal analyte processing (CAP–MAP) was used to analyse RNA cap structures by Mass Spectrometry (MS) [5, 22].

### RNA sequencing analysis

*Rnmt* and *Ram* cKO T cells were MACS magnet sorted from mouse lymph nodes using a mouse CD4 + T Cell Isolation Kit (Miltenyi Biotec), then activated for 20 h on anti-CD3 antibody (5 μg/ml) and anti-CD28 antibody (1 μg/ml) coated plates in T cell medium. CD4 T cell purities were assessed by flow cytometry and were ~98%. Cre recombinase-negative littermates (Cre-) were used as controls. RNA was isolated using Tri-reagent (Sigma/Merck) and submitted to Genewiz for strand-specific Illumina library preparation using ribosomal RNA depletion and Illumina NovaSeq paired-end 150 bp sequencing. Sequence quality was checked using FastQC. Sequences were aligned to mm10 with gencode vM25 basic annotation, and gene count tables were generated using STAR version 2.5.2b. Differential gene expression analysis was performed in R with EdgeR using a counts per million (CPM) threshold of one in at least three samples. Gene annotations were downloaded using biomaRt, plots drawn using ggplot2, and genes with an FDR below 0.05 in the differential expression analysis were submitted to Webgestalt using Wikipathways for gene set overrepresentation analysis. To assess sRNA (small RNAs) expression, reads were aligned to mm10 using STAR 2.5.2b with filters on multimapping reads removed to avoid losing data on small RNAs contained in repeating genes. Gene counts were recalculated with HTseq such that the primary alignments of multimapping reads were counted then summed read counts for each Rfam sRNA gene family were calculated. Differential expression analysis was repeated using EdgeR including the mRNA read counts to allow read count normalization and using a CPM threshold of one in at least three samples. For splicing analysis, a custom GFF annotation file, including intron and exon locations was generated using a perl script posted on seqanswers (http://seqanswers.com/forums/showthread.php?t=42420) by Alejandro Reyes and then sorted according to strand in R. Exonic and intronic reads were counted using HTSeq, and differential splicing analysis was carried out using DEXseq. Plots were drawn using ggplot2.

### Western blot

T cells were lysed in 10 mM Tris HCl pH 7.05; 50 mM NaCl; 30 mM Na_4_P_2_O_7_; 50 mM NaF; 10% glycerol; 0.5% triton X-100; 10 mM aprotonin; 1 mM leupeptin; 1 mM pepstatin; 10 mM PI2, 10 mM PI3 and 100 mM DTT on ice and sonicated 5× for 30 s at medium intensity on Bioruptor (Diagenode). Protein samples from 1 to 5 × 10^5^ cells were resolved by SDS–PAGE and transferred into PVDF membranes with Tris–glycine buffer (25 mM Tris, 190 mM glycine, 20% methanol). Membranes were incubated with primary antibodies (polyclonal anti-RNMT or anti-RAM antibodies, in-house; anti-GAPDH or anti-ACTIN antibodies, Supplementary Table S6) for 2 h at room temperature, followed by incubation with the relevant HRP-conjugated secondary antibodies (Supplementary Table S6) for 1 h at room temperature. Signals were developed using Pierce Super Signal ECL (Thermo Fisher Scientific) and visualized digitally using Fuji Las 4000 (Fujifilm).

### Translation assay

For assessing the efficiency of translation, *ex vivo* activated T cells from control and *Ram* cKO mice, collected on Day 1 and Day 2, were incubated with 1 μg/ml Puromycin for 10 min at 37°C in T cell culture medium, stained with Fixable Viability Dye eFluor 780 (eBioscience), fixed with 1% PFA in PBS (Santa Cruz Biotech) on ice, permeabilized and stained using anti-Puromycin antibodies (Millipore) and other surface markers (as above) in saponin buffer (PBS + 5% FBS + 0.1% saponin).

### Statistics

The statistical significance of the results presented was calculated using double-sided, unpaired Student’s *t*-test. Statistical analysis in bioinformatics described in RNA sequencing analysis.

## Results and discussion

### The RNMT cap methyltransferase activator, RAM, is upregulated during T cell activation

**Figure 1:**
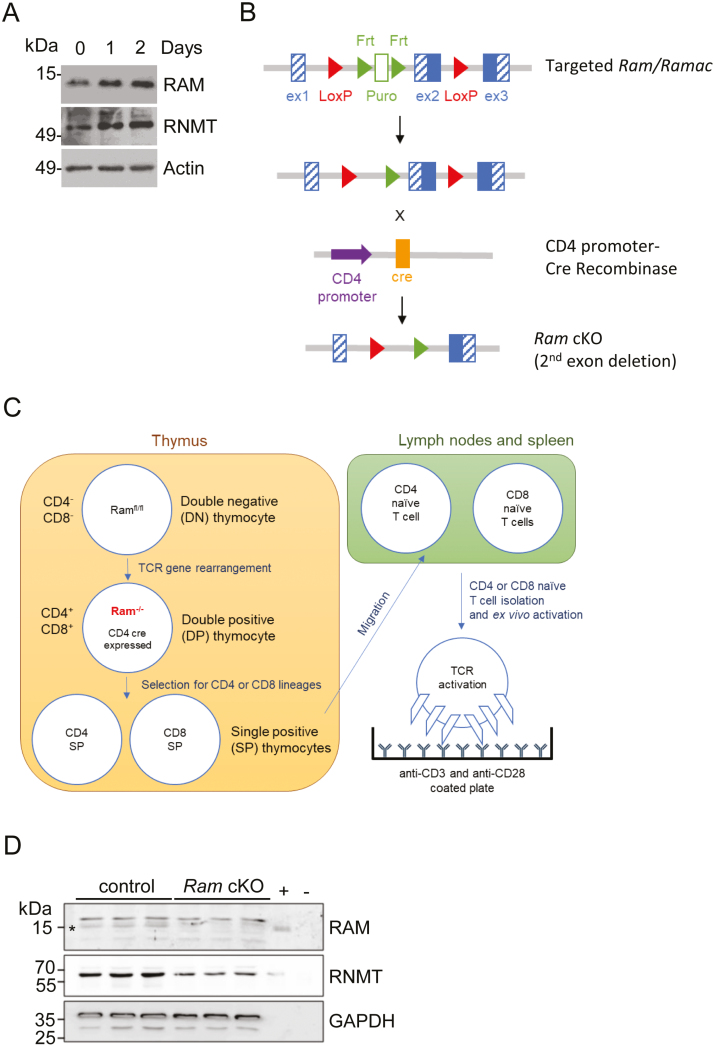
RAM and RNMT are upregulated following T cell activation. (**A**) Western blot analysis of RAM and RNMT protein levels in naïve (Day 0), and *ex vivo* activated (Days 1 and 2) CD4 T cells. ACTIN was used as a loading control. Data was generated from the cells of one mouse and is representative of data from three mice. (**B**) Schematic of *Ram* cKO strategy. (**C**) The *Ram* cKO T cell model: progenitor and mature T cell populations and *ex vivo* activation protocol are depicted. *Ram* deletion is in the double positive (DP; CD4 + CD8+) thymocytes. (**D**) Western blot analysis of RAM and RNMT protein levels in control (*Ram*^*fl/fl*^) and *Ram* cKO activated CD4 T cells. Sample in each lane generated from cells of one mouse. RNMT immunoprecipitates (+) from mouse brain lysates and (–) from liver lysates were used to verify the migration of RAM. GAPDH was used as a loading control. A star indicates the RAM band

We investigated the mechanisms by which gene expression is increased during T cell activation. Following activation of CD4 T cells *ex vivo*, RNMT and its activating subunit RAM were upregulated (Fig. 1A). RAM and RNMT were also found to be upregulated on CD4 and CD8 T cell activation in the ImmPRess mass spectrometry data set [23]. To study the role of RAM in T cells, we deleted the *Ram* gene (*Fam103a1*/*Ramac*) at the double positive (DP) stage of T cell development. Mice with floxed *Ram* alleles were crossed with mice expressing CD4 promoter-driven Cre recombinase (CD4-Cre) to generate *Ram*^fl/fl^ CD4-Cre mice (*Ram* cKO, conditional knockout, Fig. 1B and C) [21]. *Ram* gene deletion occurs in CD4 + CD8 + DP cells, the developmental precursors of CD4 and CD8 single positive (SP) T cells (Fig. 1C). Loss of RAM protein was confirmed in *Ram* cKO CD4 T cells (Fig. 1D). In activated *Ram* cKO CD4 T cells there was also decreased RNMT protein, consistent with the dependency of RNMT expression on RAM protein observed in other cell lineages and cell lines (Fig. 1D) [15, 19, 24].

### *Ram* is required for T cell development and activation

**Figure 2:**
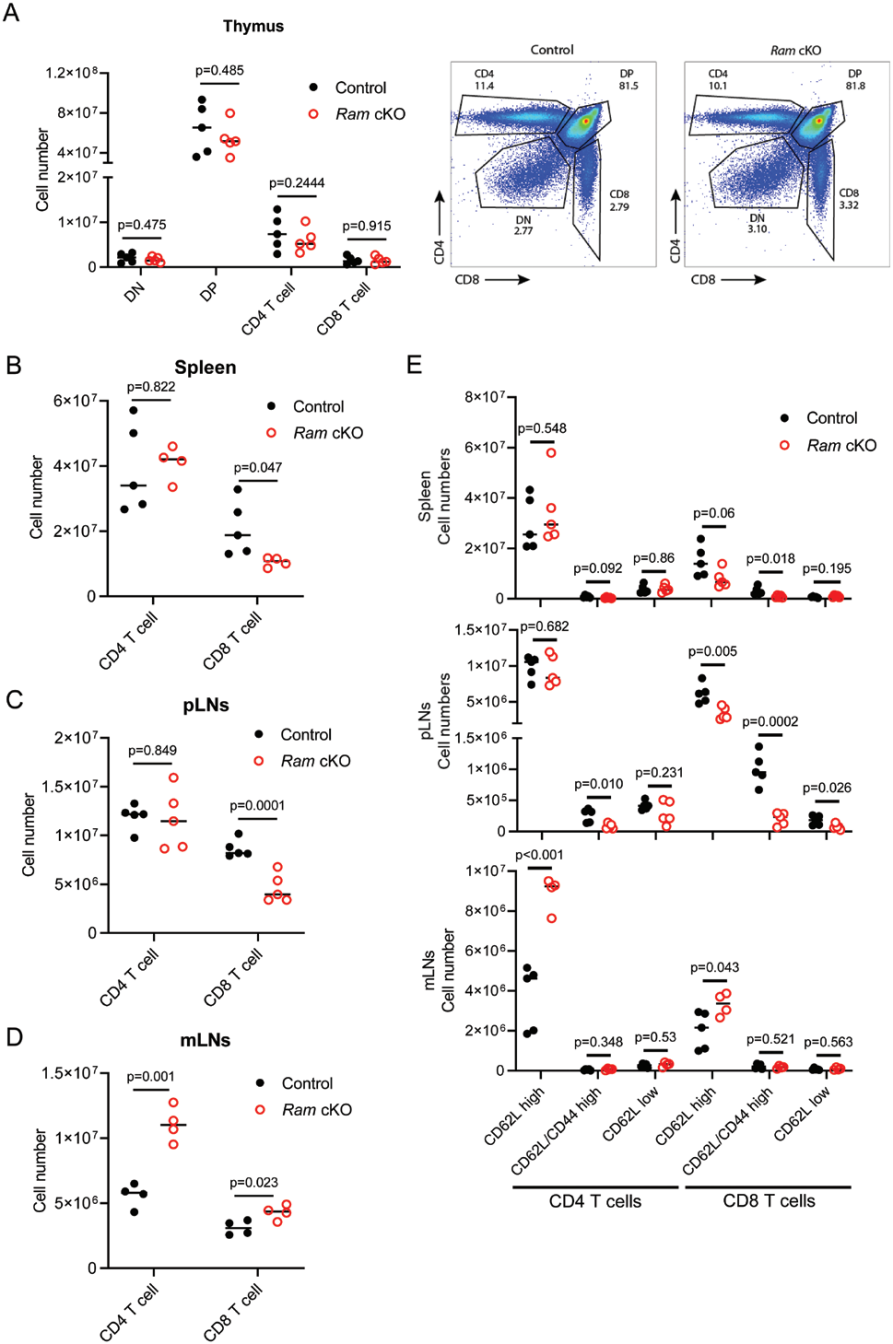
*Ram* deletion results in a reduction in CD8 T cells. (**A**) A number of CD4 and CD8 double negative (DN), double positive (DP) and single positive T cells in the thymus in control (*Ram*^*fl/fl*^, *n* = 5) and *Ram* cKO (*n* = 5) mice. Representative FACS plot, right panel, including % in each gate. Number of CD4 and CD8 T cells in (**B**) spleen (control (*n* = 5) and *Ram* cKO (*n* = 4) mice), (**C**) peripheral lymph nodes (pLNs) (control (*n* = 5) and *Ram* cKO (*n* = 5) mice) and (**D**) mesenteric lymph nodes (mLNs) (control (*n* = 4) and *Ram* cKO (*n* = 5) mice). (**E**) Percentage of CD4 and CD8 CD62L high, CD62L/CD44 high and CD62L low T cells in spleen (top panel, control (*n* = 5) and *Ram* cKO (*n* = 5) mice), peripheral lymph nodes (pLNs, middle panel, control (*n* = 5) and *Ram* cKO (*n* = 5) mice), and mesenteric lymph nodes (mLNs, bottom panel, control (*n* = 5) and *Ram* cKO (*n* = 4) mice). Dots indicate biological replicates, lines indicate means, and *P* values from Student’s *t* test. Gating strategies are depicted in Supplementary Figs. 1 and 2

*Ram* cKO DP, CD4 SP, and CD8 SP thymocytes were similar in number to controls (Fig. 2A). In the spleen and peripheral lymph nodes, the number of *Ram* cKO CD8 T cells was reduced whereas the number of *Ram* cKO CD4 T cells was unchanged (Fig. 2B and C). Previously in *Rnmt* cKO (*Rnmt*^fl/fl^ CD4-Cre mice), the number of CD8 and CD4 T cells was observed to be reduced in the spleen and lymph nodes, with a greater reduction in CD8 T cells [5]. Therefore, the deletion of *Rnmt,* the cap methyltransferase catalytic subunit, has a more severe impact on development than the deletion of *Ram,* the cofactor. In the mesenteric lymph nodes (mLNs), *Ram* cKO CD4 and CD8 T cells were increased (Fig. 2D). Increased T cells in the mLN were also observed in the *Rnmt* cKO (data not shown). The mechanism behind the increased cellularity of the mLNs is unknown but might involve mild inflammation and increased recruitment of immune cells. Consistent with this, *Rnmt* cKO and *Ram* cKO mice occasionally develop anal prolapses (~5% incidence). We speculate that this phenotype may relate to reduced T-regulatory cell function which could reduce tolerance to gut antigens and affect gut epithelial homeostasis [25]. The impact of *Ram* cKO on naïve, central memory, and effector memory CD4 and CD8 T cells was assessed by analysing CD62L and CD44 expression (Fig. 2E). Most splenic and lymph node T cells in uninfected mice are expected to have a naïve phenotype and this was the case in both control and *Ram* cKOs. The proportion of central memory CD4 and CD8 T cells (CD62L high/CD44 high), was lower in *Ram* cKOs, consistent with decreased numbers of CD4 and CD8 central memory cells in the spleen and peripheral lymph nodes.

### *Ram* is required for the proliferation of activated T cells

**Figure 3:**
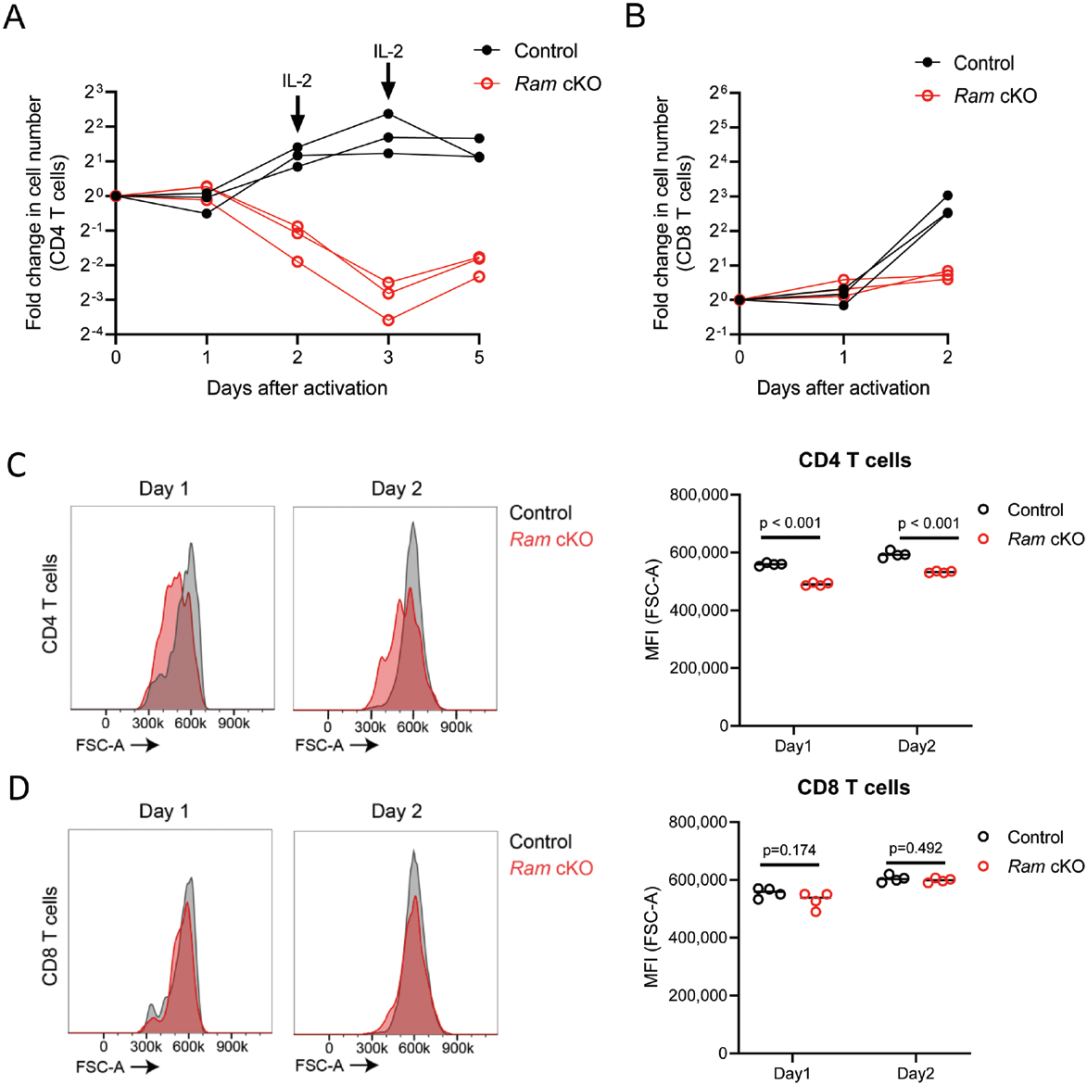
Activated Ram cKO CD4 and CD8 T cells have a proliferation defect. Splenocytes from control (*Ram*^fl/fl^) (*n* = 3) and *Ram* cKO mice (*n* = 3) were activated *ex vivo* and cultured. The number of cells was determined: (**A**) CD4 T cells and (**B**) CD8 T cells. Forward scatter (FSC-A) was measured on Days 1 and 2 in (**C**) CD4 T cells, (**D**) CD8 T cells. Representative histograms left panels. MFI plots, right panels (*n* = 4). Dots indicate biological replicates, lines indicate means, and *P* values from Student’s *t* test. Gating strategies are depicted in Supplementary Figs. 2 and 3

Following activation *ex vivo* by culturing with anti-CD3 and anti-CD28 antibodies, CD4 and CD8 T cells in spleen cell suspensions from control mice proliferated rapidly (Fig. 3A and B). *Ram* cKO CD4 and CD8 T cells failed to proliferate. Activated *Ram* cKO CD4 T cells had lower forward scatter (FSC-A), than controls, indicating reduced size (Fig. 3C). The *Ram* cKO CD8 T cell FSC was equivalent to controls (Fig. 3D). In *Rnmt* cKO, CD4 and CD8 T cells also had reduced FSC and proliferation, therefore following activation *Ram cKO* T cells exhibited a similar but milder phenotype to *Rnmt* cKO ( [5], data not shown).

**Figure 4:**
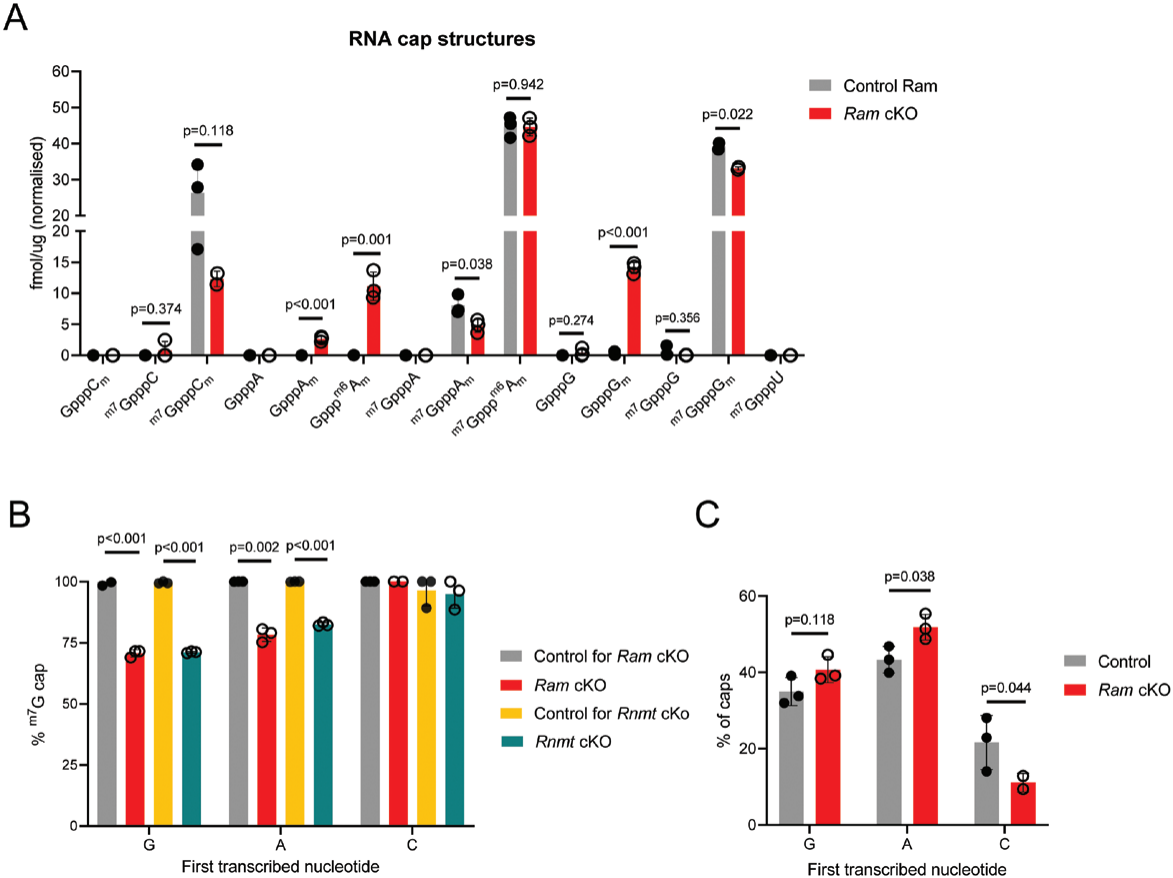
Ram cKO CD4 T cells have reduced ^m7^G-capped RNA. CD4 T cells were purified from control (*n* = 3), *Ram* cKO (*n* = 3) and *Rnmt* cKO (*n* = 3) mice and activated *ex vivo* for 26 h. (**A**) CAP–MAP quantitation of cap structures in control and *Ram* cKO CD4 T cell mRNA. Note C cap detection failed in one *Ram* cKO replicate. (**B**) The proportion of ^m7^G-capped mRNAs initiating with guanosine (G), adenine (A) or cytosine in *Ram* cKO and *Rnmt* cKO CD4 T cells. (**D**) The proportion of capped mRNAs initiating with guanosine (G), adenine (A), or cytosine (C) in control and *Ram* cKO CD4 T cells. Dots show biological replicates, bars indicate mean, and *P* values from Student’s *t* test

Since RAM increases RNMT-dependent ^m7^G cap methylation, we investigated the impact of *Ram* deletion on RNA cap structures [8, 15, 17]. CAP–MAP mass spectrometry was used to quantitate RNA cap structures in CD4 T cells (Fig. 4) [22]. As previously reported, wild-type T cells have a ^m7^G cap structure on almost all mRNA (Fig. 4A) [5, 22]. In *Ram* cKO CD4 T cells, the mature caps ^m7^GpppC_m_, ^m7^GpppA_m_, and ^m7^GpppG_m_ were decreased (Fig. 4A). Consistent with the loss of guanosine cap methylation (^m7^G) some of the corresponding precursor immature caps, GpppA_m_, Gppp^m6^A_m_, and GpppG_m_ were increased (Fig. 4A). When the initiating transcribed nucleotide was G or A, the corresponding ^m7^G caps ^m7^GpppG_m_ and ^m7^GpppA_m_ were reduced by ~25% in *Ram* cKO and *Rnmt* cKO CD4 T cells (Fig. 4B). When the first transcribed nucleotide was cytosine, the proportion of ^m7^G caps remained high in *Ram* and *Rnmt* cKO CD4 T cells, consistent with GpppC_m_ and other immature C caps being unstable (Fig. 4B). As a consequence, *Ram* cKO T cells had a lower proportion of mRNAs initiating with cytidine (Fig. 4C), and a similar observation was made in *Rnmt* cKO [5].

### *Ram* controls the expression of mRNA and snoRNA associated with ribosome biogenesis

**Figure 5:**
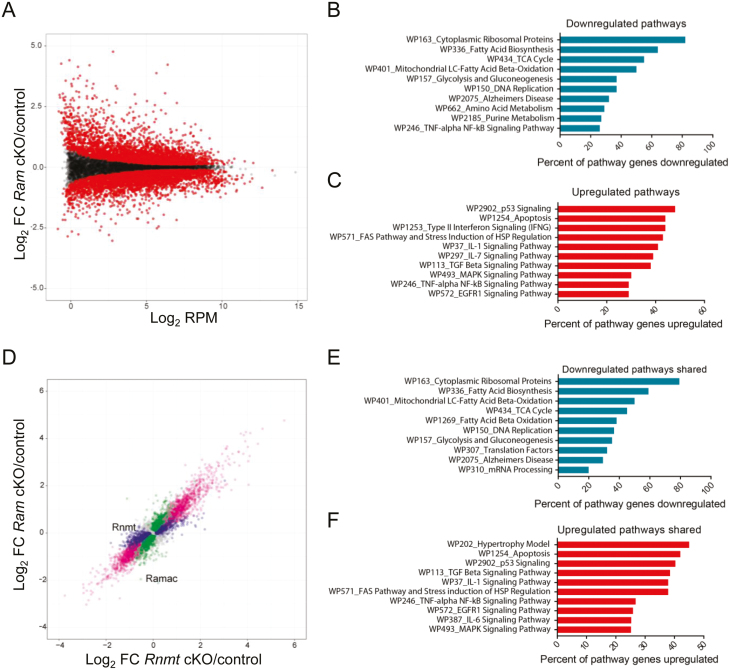
A comparison of the RNMT and RAM-regulated transcriptomes. (A) MA plot of RNA expression in control (*Ram fl/fl, n* = 3) and *Ram* cKO (*n* = 3) activated CD4 T cells. Dots represent genes. Reads per million mapped reads (RPM) on the *x*-axis. Control and *Ram* cKO samples were compared using the EdgeR exact test and adjusted *P* value used. Pathway analyses (FDR < 30%) of significantly (**B**) downregulated or (**C**) upregulated *Ram* cKO target RNAs (adjusted *P*-value < 0.05). (**D**) Comparison of RNA level changes in *Ram* cKO (*n* = 3) and *Rnmt* cKO (*n* = 3) activated CD4 T cells. Equivalently regulated genes, pink; regulated in *Ram* cKO only, green; regulated in *Rnmt* cKO only, blue. Pathway analyses of *Ram* cKO and *Rnmt* cKO shared target RNAs (FDR < 30%), (**E**) downregulated or (**F**) upregulated (adjusted *P* value < 0.05)

Since *Ram* cKO T cells had reduced proliferation, we investigated changes in their transcriptome which may be consistent with this defect. *Ram* cKO CD4 T cells were activated *ex vivo* for 20 hours and the transcriptomes were analysed by RNA sequencing. The sequencing performed here will not distinguish the mechanism of gene regulation and whether RAM has a direct or indirect impact, however, all genes regulated will potentially contribute to the phenotype observed. Out of 13140 genes whose RNAs passed detection thresholds, 3496 were significantly reduced and 3294 were significantly upregulated in the *Ram* cKO CD4 T cells compared to controls (Fig. 5A, Supplementary Table S1). Consistent with a defect in cell growth and proliferation, gene set overrepresentation analysis indicated that RNAs downregulated in *Ram* cKO CD4 T cells were highly enriched for transcripts encoding ribosomal proteins, with over 80% of genes in this pathway being sensitive to *Ram* deletion (Fig. 5B, Supplementary Table S1). Other downregulated RNAs in the *Ram* cKO T cells belonged to gene families associated with metabolism, amino acid biosynthesis pathways, TCA cycle and DNA replication pathways. Within the group of upregulated genes in the *Ram* cKO cells, there were genes encoding proteins involved in signalling or apoptosis (Fig. 5C, Supplementary Table S1).

Since deletion of *Ram* in T cells was having a similar but milder phenotype to that observed on the deletion of *Rnmt*, we investigated if a subset of *Rnmt-*dependent genes is also *Ram* dependent. The regulation of RNAs in *Ram* cKO and *Rnmt* cKO CD4 T cells had a high correlation (Fig. 5D–F, Supplementary Tables S2 and S5). The transcripts of 2907 genes were repressed in both *Ram* cKO and *Rnmt* cKO CD4 T cells, 589 genes were repressed only in *Ram* cKO and 607 were repressed only in *Rnmt* cKO. Two thousand six hundred and sixty eight genes were increased in both *Ram* cKO and *Rnmt* cKO CD4 T cells, 626 genes were increased in *Ram* cKO only and 599 genes were increased in *Rnmt* cKO only. Notably, no genes were significantly changed in the opposite directions in *Rnmt* and *Ram* cKO CD4 T cells. Of the genes that were responsive to both *Rnmt* and *Ram* cKOs, there was a trend towards larger fold changes in the *Rnmt* cKO.

**Figure 6:**
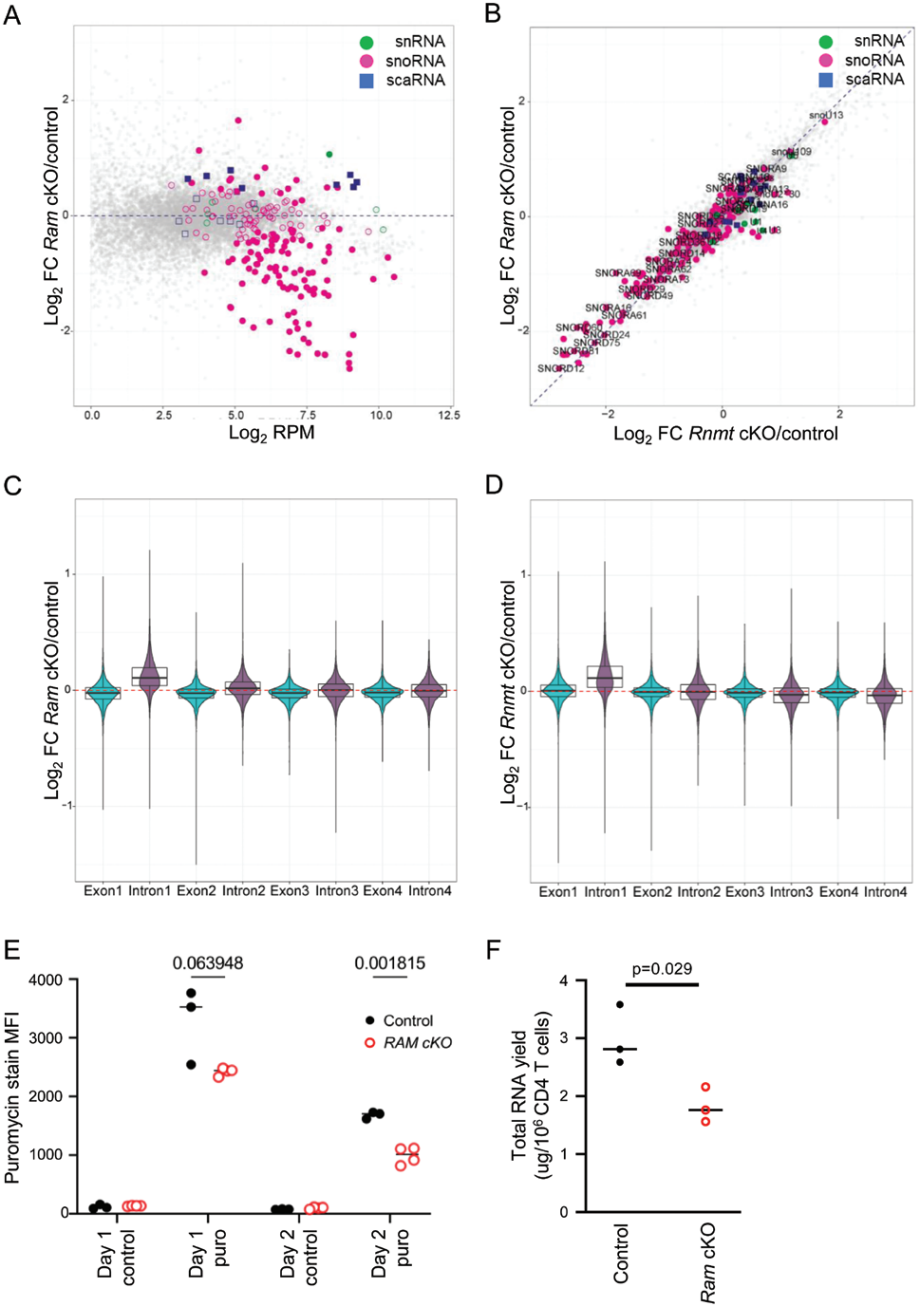
RAM is required for efficient translation following T cell activation. (**A**) Small RNA levels in *Ram* cKO (*n* = 3) and control (*Ram*^*fl/fl*^*, n* = 3) activated CD4 T cells, grouped by RFAM family. Small nuclear RNAs (snRNAs), green; small nucleolar RNAs (snoRNAs), pink; small Cajal-body specific RNAs (scaRNAs), blue. Filled dots, adjusted *P* value < 0.05. (**B**) Comparison of small RNA level changes in *Ram* cKO and *Rnmt* cKO CD4 T cells compared to controls (*Ram*^*fl/fl*^, *Rnmt*^*fl/fl*^, respectively). (**C**–**D**) Splicing analysis in activated CD4 T cells. Reads aligning to exons and introns were normalized to the total reads for the transcript and read densities for each transcript were compared between controls and (C) *Ram* cKO and (D) *Rnmt* cKO. Violin plots represent the frequency density. Box plots show median, upper and lower quartiles. Whiskers, 1.5× interquartile range. (**E**) CD4 T cells were activated *ex vivo* and translation rates were determined using puromycin incorporation into nascent peptides. Control (*n* = 3) and *Ram* cKO (*n* = 4). Cells incubated without puromycin were used for a baseline signal. (F) RNA yield from 10^6^ control and *Ram* cKO CD4 T cells activated *ex vivo* for 24 h (*n* = 3). Dots show biological replicates, bars indicate mean, *P* values from Student’s *t* test. The gating strategy is depicted in Supplementary Fig. 6

All RNA pol II transcripts have the potential to initially receive a ^m7^G cap, including the small nuclear RNAs (snRNAs), small nucleolar RNAs (snoRNAs), and small Cajal bodies-specific RNAs (scaRNAs) [26, 27]. The cap of most snoRNAs and scaRNAs is removed as they are processed from introns or precursor long non-coding RNAs. When the cap is retained on small RNAs, they receive additional cap modifications. The caps on small RNA caps are methylated by RNMT and we previously confirmed that their expression is RNMT-dependent in T cells [5]. Transcript sequencing indicated that 93 snoRNAs were decreased out of 176 detected in *Ram* cKO CD4 T cells, compared to controls (Fig. 6A, Supplementary Table S3). Similarly, in *Rnmt* cKO, 88 snoRNAs were repressed (Supplementary Table S4). In contrast, many scaRNAs were upregulated in *Ram* cKO and *Rnmt* cKO T cells when compared to control (eight and six scaRNAs respectively, out of 16 detected) (Fig. 6A and B, Supplementary Tables S3 and 4). snRNAs were mildly impacted; out of nine detected, one was increased and one was decreased in *Ram* cKO T cells and four were increased in *Rnmt* cKO activated CD4 T cells (Fig. 6A and B, Supplementary Tables S3 and S4).

Although changes in snRNA were minimal in *Ram* cKO T cells, the ^m7^G cap can also influence splicing by recruiting the Cap Binding Complex (CBC), which aids first intron removal [28, 29]. A defect in splicing of the first intron was observed in the transcriptome analysis in activated *Ram* cKO CD4 T cells (Fig. 6C), similar to the defect observed in activated *Rnmt* cKO CD4 T cells (Fig. 6D).

### RAM is required for protein synthesis in activated T cells

To investigate the functional consequences of *Ram* KO, we analysed translation rates using puromycin incorporation into nascent peptides. In *Ram* cKO CD4 T cells, puromycin incorporation was reduced indicating reduced translation, consistent with reduced expression of ribosomal proteins and ribosome biogenesis factors (Fig. 6E). In *Ram* cKO CD4 T cells, total RNA per cell was reduced, the majority of which is ribosomal RNA (Fig. 6F). Reduced ribosomal RNA is consistent with reduced expression of TAF1D, an RNAPI component, reduced UTP14a, a component of the U3 snoRNP which mediates rRNA cleavage and reduced NPM1 a ribosome biogenesis factor.

In summary, regulation of gene expression is critical to T cell function; increasing cellular protein content during activation and proliferation, and reshaping the proteome during differentiation into distinct T cell lineages. RNA cap formation has important roles in RNA expression, stability, processing, and translation. Here we describe that RAM, the cofactor for the N-7 guanosine cap methyltransferase RNMT is upregulated during T cell activation and is required for RNMT expression and function in methylating the RNA cap guanosine. On *Ram* gene deletion, T cells fail to proliferate following activation. Consistent with this defect in growth and proliferation the RNAs repressed in *Ram* cKO are members of the ribosomal gene family and the snoRNAs which mediate ribosomal RNA processing. In addition to RAM, RNMT also interacts with eIF4E and eIF4E impacts on RNA cap formation. The RNMT–eIF4E interaction may have roles during T cell activation, particularly in RNA export and translation initiation [30, 31]. The role of the other capping enzymes in T cell activation is yet to be determined.

## Supplementary Material

kyad021_suppl_Supplementary_Tables_S1-S6

kyad021_suppl_Supplementary_Figures_S1-S4

## Data Availability

RNA sequencing data is present in the GEO data series: GSE198541.

## Abbreviations

CBC: cap binding complex;
CPM: counts per million;
FBS: fetal bovine serum;
LARP1: la ribonucleoprotein 1;
MS: mass spectrometry;
PBS: phosphate-buffered saline;
RNMT: RNA guanosine N-7 cap methyltransferase;
snRNA: small nuclear RNAs;
snoRNA: small nucleolar RNAs;
scaRNA: small Cajal bodies specific RNAs;
sRNA: small RNAs.
