# Supplementary material for: RAM is upregulated during T cell activation and is required for RNA cap formation and gene expression
Source: Discov Immunol. 2023 Nov 17;3(1):kyad021. doi: 10.1093/discim/kyad021 (PMC10989996; doi:10.1093/discim/kyad021)
Supplement: kyad021_suppl_Supplementary_Figures_S1-S4 [file kyad021_suppl_Supplementary_Figures_S1-S4.pdf]

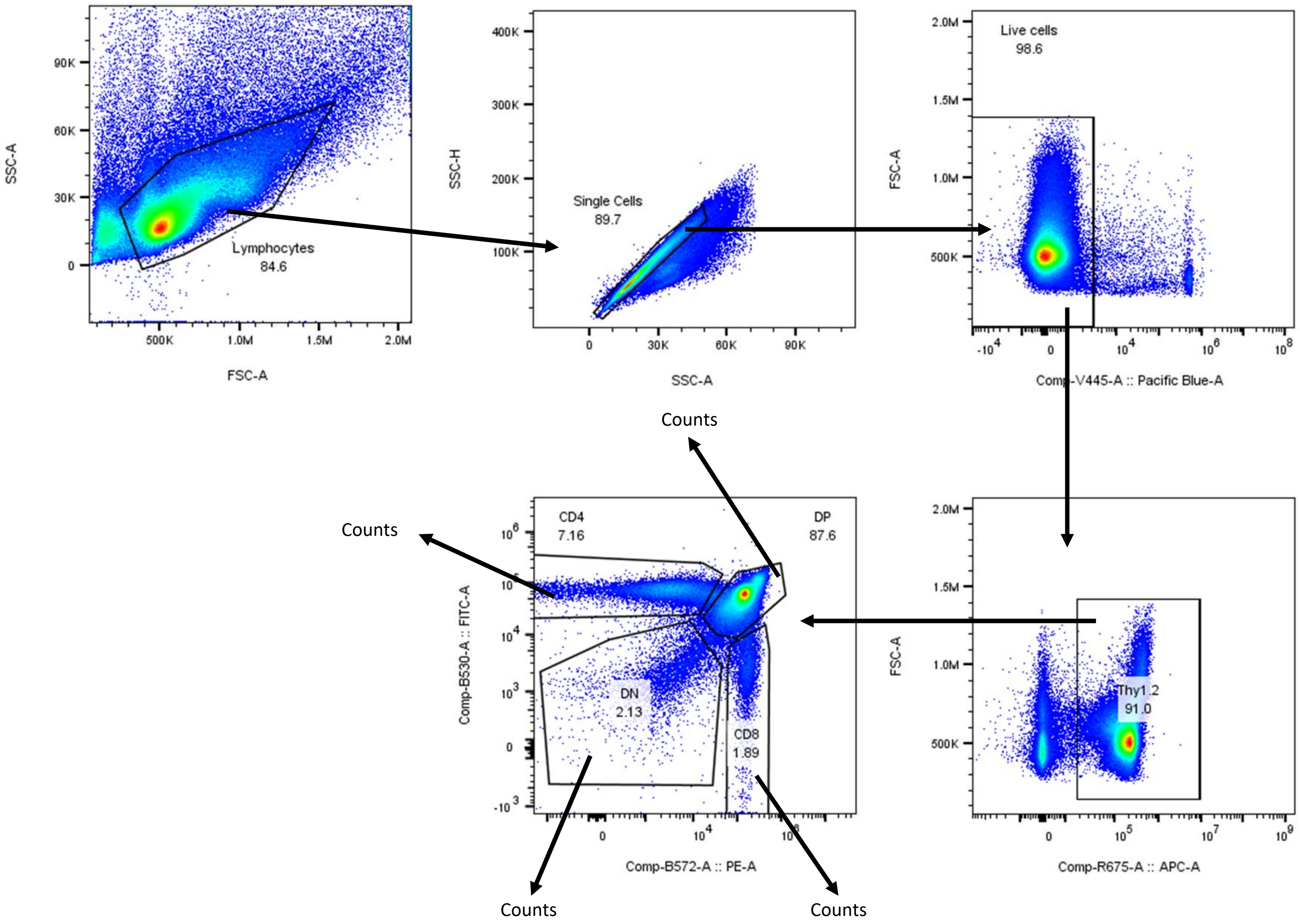

Supplementary Figure 1 (For Figure 2A)

Gating strategy for flow cytometry analysis of DN, DP, CD4 and CD8 T cell populations in the thymus. Live cells (DAPI-); T cells (Thy1.2+). Numbers indicate percentage of cells within each gate**.**

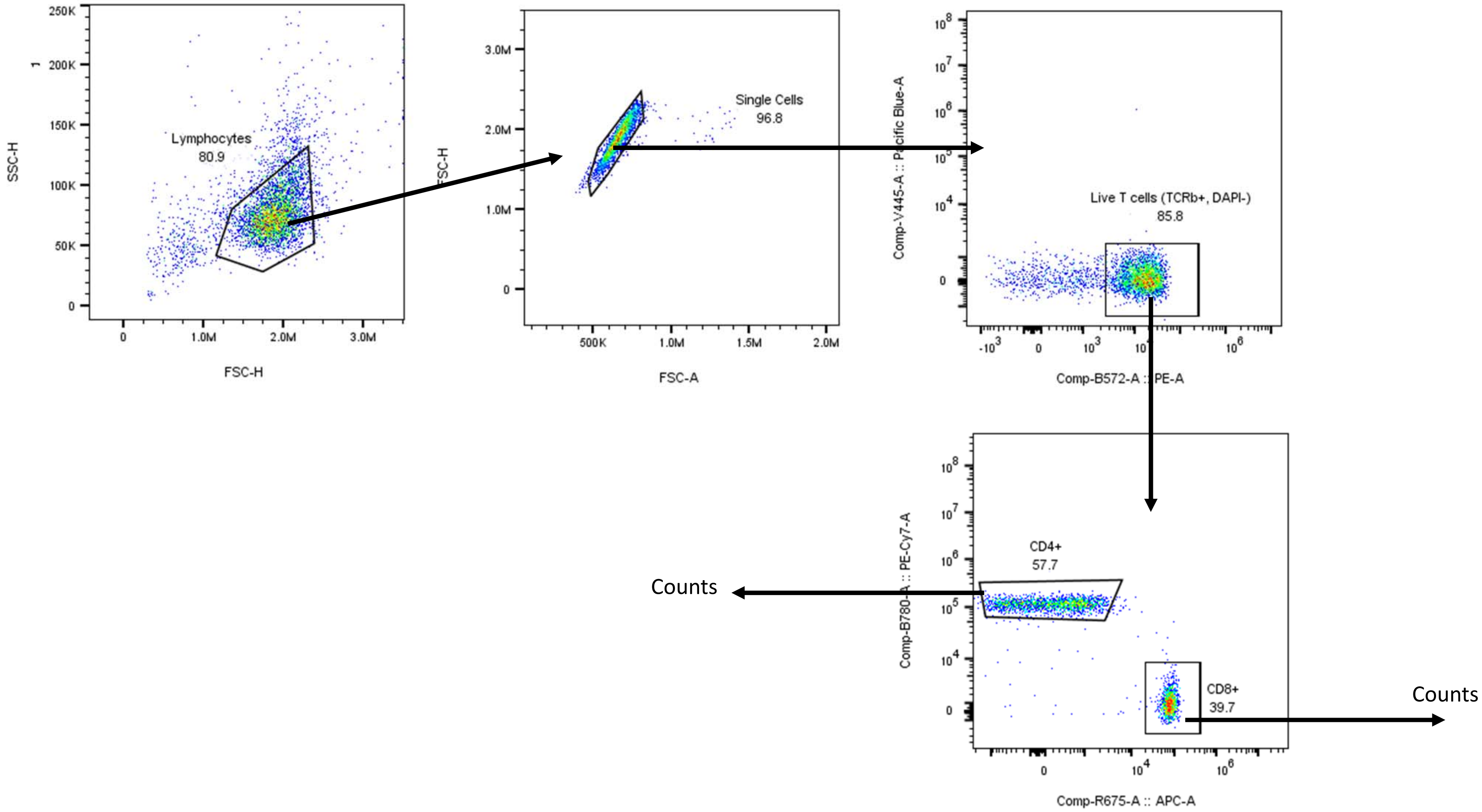

Supplementary Figure 2 (For Figure 2B-D and 3)
Gating strategy for flow cytometry analysis of CD4 and CD8 T cell populations. Numbers indicate percentage of cells within each gate.

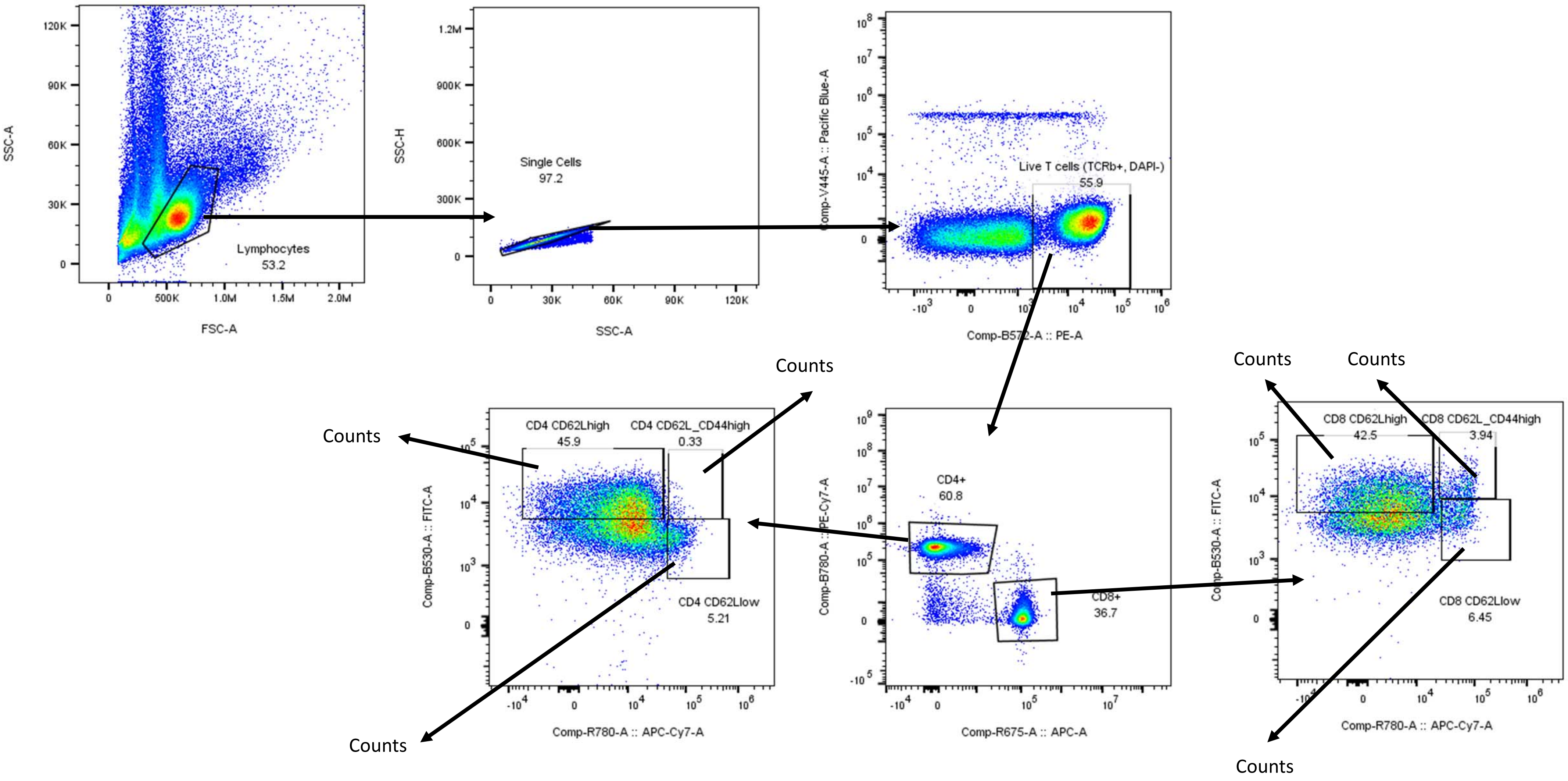

Supplementary Figure 3 (For Figure 2E)
Gating strategy for flow cytometry analysis of CD4 and CD8 T cell populations expressing CD62L/CD44 in spleen, pLNs and mLNs. Numbers indicate percentage of cells within each gate.

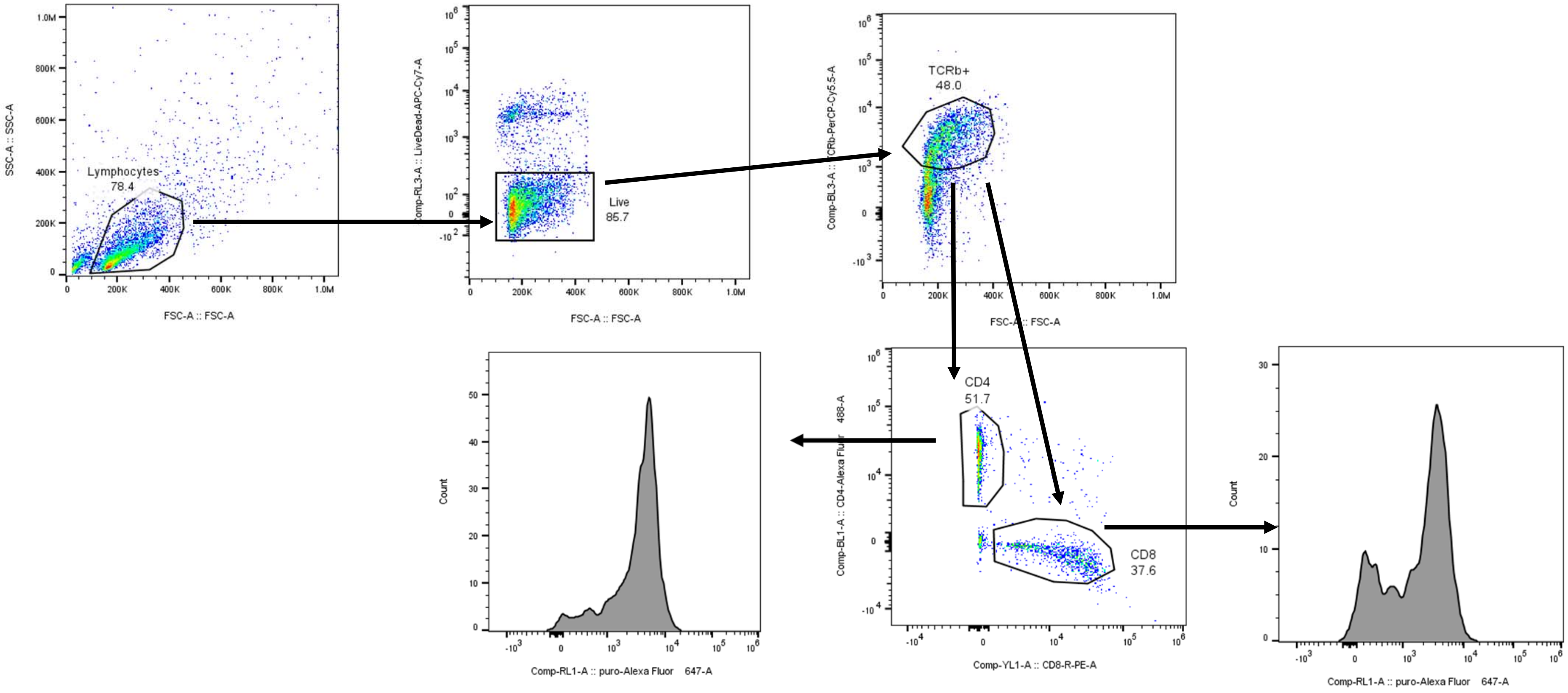

Supplementary Figure 4 (For Figure 6E)

Gating Strategy for flow cytometry analysis of puromycin incorporation in CD4 and CD8 T cells from pLNs and mLNs. Numbers indicate percentage of cells within each gate. Histograms represent the puromycin MFI for each T cell population.
